# Detecting Cyberattacks on Electrical Storage Systems through Neural Network Based Anomaly Detection Algorithm

**DOI:** 10.3390/s22103933

**Published:** 2022-05-23

**Authors:** Giovanni Battista Gaggero, Roberto Caviglia, Alessandro Armellin, Mansueto Rossi, Paola Girdinio, Mario Marchese

**Affiliations:** Department of Electrical, Electronic and Telecommunications Engineering and Naval Architecture—DITEN, University of Genoa, Via Opera Pia 11A, 16145 Genoa, Italy; giovanni.gaggero@edu.unige.it (G.B.G.); roberto.caviglia@edu.unige.it (R.C.); alessandro.armellin@edu.unige.it (A.A.); mansueto.rossi@unige.it (M.R.); paola.girdinio@unige.it (P.G.)

**Keywords:** cybersecurity, distributed energy resources, electrical battery storage systems, neural network, autoencoder, anomaly detection

## Abstract

Distributed Energy Resources (DERs) are growing in importance Power Systems. Battery Electrical Storage Systems (BESS) represent fundamental tools in order to balance the unpredictable power production of some Renewable Energy Sources (RES). Nevertheless, BESS are usually remotely controlled by SCADA systems, so they are prone to cyberattacks. This paper analyzes the vulnerabilities of BESS and proposes an anomaly detection algorithm that, by observing the physical behavior of the system, aims to promptly detect dangerous working conditions by exploiting the capabilities of a particular neural network architecture called the autoencoder. The results show the performance of the proposed approach with respect to the traditional One Class Support Vector Machine algorithm.

## 1. Introduction

The uncertainty of Renewable Energy Sources production and the high scalability of solutions such as solar panels enable the shift from a centralized production of energy to a distributed one. Consequently, electrical grids are moving toward a large use of Distributed Energy Resources that are usually referred to as small or medium-scale unit of power generation typically connected to a low or medium voltage grid. In order to achieve high energy efficiency and improve the resilience of the overall electrical system, DERs may be deployed within microgrids or energy communities. A fundamental element of microgrids is represented by Battery Energy Storage Systems (BESSs). Storage is used to balance the production of uncontrollable sources like photovoltaic systems, both for economic purposes and to allow the microgrid to operate in islanded mode. In this context it is necessary to use a control system exploiting either Supervisory Control and Data Acquisition (SCADA) or Distributed Control Systems (DCS). SCADA solutions present different vulnerabilities that may depend on the used communication protocol such as IEC 61850 or Modbus. The geographic location of devices may further pose a problem when implementing basic physical security countermeasures. For these reasons, if an attacker gains access to the control network, consequences can be very serious. Following the paradigm of defense in depth, solutions to monitor security are fundamental tools to improve the resilience of the grid towards cyberattacks.

The main contribution of this work is the development of an anomaly detection algorithm based on a neural network autoencoder. The anomaly detection algorithm aims to substitute the action of a human operator, rapidly detecting possible dangerous working conditions and promptly implementing countermeasures. Moreover, we analyze the architecture of a storage system within a microgrid, in order to evaluate the whole attack surface and the associated risk. In the remainder of the paper, anomaly is defined as an improper working condition of the system. From the point of view of data receivers, anomaly can be associated both with an abnormal working condition and with data manipulation by attackers.

The paper is structured as follows. Section 2 analyzes the state of the art of DERs and BESS vulnerabilities, as well as related security monitoring systems. Section 3 presents the structure of a BESS within a microgrid environment, also evidencing common vulnerabilities and associated risks. Section 4 describes the proposed anomaly detection algorithm based on an autoencoder. Section 5 presents the simulation environment developed in order to test the proposed solution. Section 6 shows the results of the performance evaluation of the proposed approach. Section 7 discusses the results and Section 8 draws the conclusions.

## 2. State of the Art

Electrical SCADA systems are mostly based on industrial protocols, such as Modbus and IEC 61850, which lend themselves to severe vulnerabilities [1]. The main issue is that they lack encryption and authentication, so they are prone to Man In The Middle (MIIT) attacks [2,3]. An evaluation of attack scenarios against DERs, a systematic DER resilience analysis methodology, as well as quantifiable resilience metrics and design principles, are proposed in [4]. Authors in the paper [5] list possible vulnerabilities of DERs and propose basic functionalities for risk mitigation. The effects of cyber attacks on a supercapacitor-based energy storage in a Hybrid Power System are investigated in [6]. Ref. [7] discusses potential cyber-attack schemes and defense strategies within IoT-enabled BMS systems. An analysis of physical and cyber threats that afflict BESS is proposed in [8]. A comprehensive overview of vulnerabilities of battery management systems and the application of Blockchain-based solutions to address these issues are presented in [9]. Several papers propose novel control strategies for BESS to limit the impact of cyber attacks in microgrid environments [10,11].

Anomaly detection techniques are widely used in power systems and, in particular, in distributed generation and microgrids [12]. A review of recent detection algorithms is reported in [13]. A compilation of intrusion detection and prevention systems, specifically designed for smart grid environments, is included in [14]. Authors in the paper [15] propose an anomaly detection algorithm to identify the attacks on Photovoltaic (PV) systems, such as PV disconnection from the grid, power curtailment, volt-var attack, and inversion of the power flow in a portion of the distribution grid with a sufficient percentage of DER penetration, by exploiting semi-supervised ML algorithms like Neural network autoencoder, One Class Support Vector Machine, Isolation Forest, Random Forest with synthetic corruption, Principal Component Analysis (PCA) with convex hulls, and Inverse-PCA technique. Ref. [16] shows a contextual anomaly detection method based on an artificial neural network and explains the use of this method to discover voltage control manipulation in the low voltage distribution grid. Ref. [17] proposes a high-dimensional data-driven cyber-physical attack detection and identification approach that is based on data measured by electric waveform sensors in power distribution networks and on the use of statistical leverage scores. Malicious actions on DERs can be pursued by different methods. While many papers focus on network attacks, Ref. [18] shows firmware modification attacks to solar inverters, evidencing the relative impact on a simulated microgrid architecture, and also proposing a ML-based algorithm to detect such types of malicious actions.

A large group of scientific studies have shown that autoencoder architectures are effective for fault and anomaly detection and can outperform linear Principal Component Analysis (PCA) and Kernel PCA [19]. A deep learning scheme composed of Long Short Term Memory-Stacked Autoencoders and Convolutional Neural Network (CNN-SAE) followed by a softmax activation layer has been used for fault detection in a wind turbine in Ref. [20]. Three different autoencoding schemes (multilayer perceptron, convolutional, and long-short term memory) for fault detection are used in Ref. [21]: features extracted from measured signals feed the neural network; the classification is based on a threshold on the reconstruction error. An approach based on a fully-connected neural network autoencoder to detect cyberattacks within a photovoltaic system, similarly to the scheme proposed in this paper for storage systems, has been suggested by the same authors in Refs. [22,23].

## 3. Attack Surface of Storage Systems

### 3.1. Use Case Scenario

Storage Battery systems are composed of different electric, electronic, and communication devices. We consider a typical scenario of a storage system connected to a microgrid controlled by a SCADA system. From the electrical point of view, it is composed of:

- The modules of cells (one or more), equipped with their own Battery Management System (BMS) which ensures to maintain the correct safe range in terms of voltage, current, temperature, and other physical parameters;
- DC/DC converter, which is an electronic converter adapting the voltage of the cells to the voltage suitable for the Active Front End (AFE);
- Active Front End, which is an electronic converter transforming direct current into a three phase alternating current, allowing bidirectional power flow.

The BMS is an electronic system that manages a rechargeable battery (cell or battery pack); its main tasks are: protecting the battery from the unsafe operating area; monitoring its state; calculating secondary data; reporting data; controlling its environment; and authenticating and/or balancing it. BMS can communicate to a higher-level controller through different solutions, such as different serial communication solutions, CANBus, Modbus, and even through specific protocols and gateways in series.

**Figure 1:**
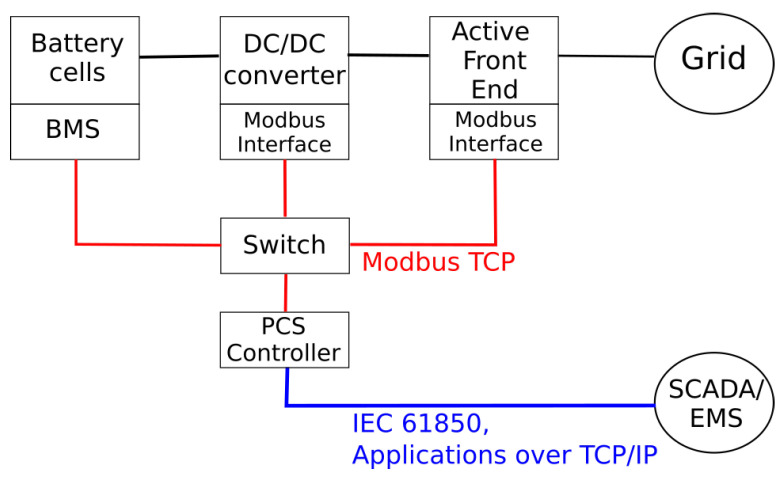
Typical architecture of a BESS within a smart microgrid.

The same communication protocols can be used by power electronic converters in order to communicate between them and with a Process Control System (PCS) usually implemented by an industrial PC, which acts as an interface between the SCADA system and local controllers. PCSs use a local Human Machine Interface (HMI) that allows the interaction with monitoring and control functions. The communication between PCS and SCADA systems can be based on protocols belonging to the IEC 61850 suite. The overall scheme is shown in Figure 1.

SCADA systems communicates with PCSs for monitoring and control purposes. Microgrids are usually equipped with an Energy Management System (EMS). The role of the EMS is to plan the energy production of dispatchable sources. Storage systems can usually operate in three different modes:

- P/Q mode, where the generators inject the desired active and reactive power;
- Islanded mode, where the generators maintain the voltage and the frequency constant while providing the necessary power to balance the loads;
- Droop equations mode, where the frequency and the voltage maintained by the generators depend on the injected power.

The SCADA system has the role of setting the operational mode and, depending on that, of giving the power setpoints to the generator. A change of operational modes is needed whenever the microgrid switches between islanded and grid-connected mode.

Many different architectures can connect electronic converters to the SCADA system. Nevertheless, the proposed architecture considers a minimal scheme, with a low number of connections and no direct connection between the control device and the SCADA network, so as to reduce the attack surface.

### 3.2. Attack Model

We can categorize the possible attacks on the proposed infrastructure from different points of views.

**Aim of the attack**: it is possible to target the storage system to damage its devices, or to use the storage to cause problems to the microgrid, both from safety and economic perspectives. The control of a generator can cause severe damage to the grid, depending on the size of the generator, the features of the grid, and the operation mode: if the microgrid operates in islanded mode with only one storage system that has the role of balancing the powers, it is obvious that compromising the generator would cause a complete blackout of the microgrid. In case the power of the storage system is not essential, e.g., the microgrid has multiple generators for voltage and frequency control or the microgrid is grid-connected, compromising that the generator does not cause immediate blackout even if the damage may be relevant. For example, a voltage or frequency variation could imply the trigger of some electrical protection, or even more protection in series;

**Exploited vulnerability**: if the attacker gains access to the control network, he can first compromise the communication between the PCS and SCADA system by exploiting IEC 61850 protocols, which are prone to different types of attacks, such as Man In the Middle [2]; in that case, he would be able to send fake commands to the PCS or fake measures to the SCADA system. Moreover, the PCS can expose different services such as web applications, Virtual Network Computing software, and so on. Several works broadly analyzed the vulnerabilities of web server and common attacks such as SQL injection [24], cross-site scripting [25], broken authentication and session management [26], and Denial of Services [27]. Furthermore, the patch management in ICS environment is more complicated than in the IT sector, so those vulnerabilities persist on industrial devices. If an attacker is able to take control of the PCS through previous mentioned vulnerabilities, he would be able to communicate directly with electronic controllers and especially with the BMS, potentially causing the destruction of the whole storage system. It is worth mentioning that, even if it is a borderline case, an attacker could violate some physical security countermeasures by directly accessing the telecommunication network, since the microgrid devices can be geographically located over a wide area, making it difficult to guarantee the full protection of some devices;

**Sophistication of the attack**: given the complexity of a microgrid, even a simple attack can cause severe problems. Let us consider a bad data injection on the value of the State Of Charge (SOC) that the PCS communicates to the SCADA controller: this action can cause an erroneous programming of the EMS that would suggest wrong power setpoints. If the storage uses automatic actions when the SOC reach dangerous levels, implications would be economic, otherwise the safety of the entire system may be in danger.

A complete taxonomy of possible attacks on a storage system is not feasible, because of the dependence on many factors, including the characteristics of the grid to which the storage system is connected. Still, the proposed evaluation of the attack model suggests that different security monitoring systems working in parallel would be useful to limit risks.

## 4. Proposed Approach

We propose an anomaly detection algorithm aimed at automatically analyzing the data generated by the storage system in order to detect anomalous physical behaviors. The algorithm takes all electrical measures generated by the devices as input and returns a classification of the correctness of the behavior. In this context, an anomaly can be represented both by an abnormal behavior of the generators, and by abnormal measures received as input, such as, for example, a set of measures that are physically incompatible with each other.

The algorithm exploits the capabilities of a particular neural network (NN) architecture called autoencoder, which learns to reproduce its input after a compression of the data. The basic idea is that, after a training phase is performed by using a dataset containing only “normal” data, the NN learns to reproduce normal data with lower error and abnormal data with higher error as used and detailed in [22].

### 4.1. Procedure

The algorithm is formalized as follows.

We define the state vector $x\left(t\right)=x_{1}\left(t\right),x_{2}\left(t\right),\ldots ,x_{n}\left(t\right)$ as a vector whose elements (also called features) are the measures extracted from the system at a certain time *t*. The state vector represents the state of the system at time *t*. We periodically collect the measures representing the correct behavior of the system, so composing a training dataset ${x^{\prime }}^{TR}$ which contains the set of measures collected at given time in each row and a single type of measure collected over time in each column. We also compose a test dataset ${x^{\prime }}^{TEST}$ which contains vectors representing both good and bad behaviors, labeled correspondingly. Considering the n-th feature of ${x^{\prime }}^{TR}$, we compute the mean value ${\bar{x}}_{i}^{TR}$ as in (1) and the standard deviation $\sigma_{i}^{TR}$ as in (2), for the training dataset.

(1) $${\bar{x}}_{n}^{TR}=\frac{\sum_{k=1}^{T}{x^{\prime }}_{n}^{TR}\left(t_{k}\right)}{t_{T}-t_{1}}$$

(2) $$\sigma_{n}^{TR}=\sqrt{\frac{\sum_{k=1}^{T}{\left({x^{\prime }}_{n}^{TR}\left(t_{k}\right)-{\bar{x}}_{i}^{TR}\right)}^{2}}{t_{T}-t_{1}}}$$

Then we normalize each single measure of the training dataset as in (3):

(3) $$x_{i}^{TR}\left(t_{k}\right)=\frac{{x^{\prime }}_{i}^{TR}\left(t_{k}\right)-{\bar{x}}_{i}^{TR}}{\sigma_{i}^{TR}}$$

During the test phase, each test state vector at generic instant $t_{k}$ is normalized by using the mean value and standard deviation of the training dataset, as shown in (4)

(4) $$x_{i}^{TEST}\left(t_{k}\right)=\frac{{x^{\prime }}_{i}^{TEST}\left(t_{k}\right)-{\bar{x}}_{i}^{TR}}{\sigma_{i}^{TR}}$$

Each vector is sent one by one to the neural network, which reconstructs the input after compressing data. We define the reconstruction error as in

(5) $$e\left(t_{k}\right)=\frac{1}{n}\sum_{i=1}^{n}{\left(x_{i}\left(t_{k}\right)-{\tilde{x}}_{i}\left(t_{k}\right)\right)}^{2}$$

During the training phase, the neural network sets its parameters to minimize the error of the training dataset.

Once the neural network is trained, we feed the autoencoder with the training dataset to analyze the distribution of the reconstruction errors. This operation allows to set a threshold *E* for the reconstruction error, which will be used during the test phase in order to classify new data.

(6) $$\begin{array}{c} e^{TEST}\left(t_{k}\right)>E\to anomaly\\ e^{TEST}\left(t_{k}\right)<E\to normal \end{array}$$

**Figure 2:**
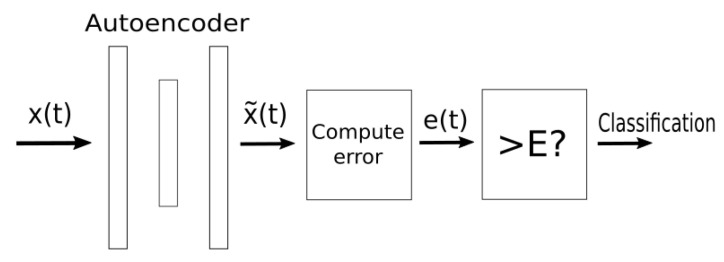
Classification scheme.

The entire classification structure is shown in Figure 2.

### 4.2. Autoencoder Architecture

The proposed neural network architecture is composed by three layers.

- Input layer, whose dimension is the same of the size of the state vector (it is fixed to 20, for this paper);
- Compressed (hidden) layer, whose dimension can vary, and, in this case, is fixed to 15;
- Output layer, which is a fully-connected layer whose dimension is the same of the input layer (20, in this case).

We chose the dimension of the hidden layer as 75% of the one of the input and output layer. Changing the range of the dimension in a reasonable way does not significantly affect the results in such type of data [22]. The first two layers utilize a ReLu activation function, while the output layer has a linear activation function. The batch size has been fixed to 256: from one side, too large of a batch size will lead to poor generalization while, on the other hand, using a smaller batch model does not guarantee to converge to the global optimum; the chosen value has been considered a good trade-off. The number of epochs will be fixed during the performance evaluation by observing the pattern of the losses over the epochs.

## 5. Materials and Methods

**Figure 3:**
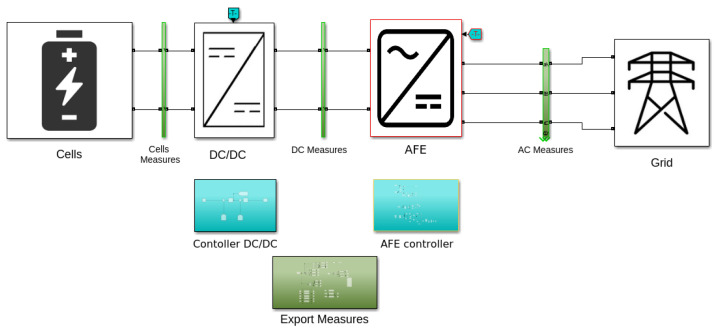
Simulink scheme implementing the used storage system.

We developed a simulator of storage systems composed of 6 arrays of cells, a DC-DC boost converter and an active front end inverter (AFE) both with their proper controller. The AFE is connected to the main grid. We use MATLAB/Simulink software and the related library Simscape. The model is electromagnetic. The control is composed of a simple feedback loop that controls the DC-DC converter by maintaining the voltage at the DC link constant and of a classic control of the inverter based on Park transformation. The overall Simulink scheme is shown in Figure 3.

We extract a series of measures from the model, which compose the state vector as discussed in Section 4. We extract all the measures at the same time and we repeat this operation with a fixed sampling time of 1 second. The composition of the state vector is shown in Table 1.

We run the simulator so as to mimic many working hours and different working conditions regarding injected powers, state of charge and other parameters of the main grid, such as small variations of voltages and frequencies. Then we extract the dataset from the Simulink software. The classification is subsequently done offline. The software is implemented in python using the Keras library [28].

The training dataset contains data related to 6 working hours where the battery completely charges and discharges. The samples are stored at each second, resulting in 21,600 samples. It only contains data related to the normal behavior of the system. All test datasets contain data of a few minutes of work under different conditions and, unlike the training dataset, contain both data related to normal and abnormal behavior.

## 6. Performance Evaluation

**Figure 4:**
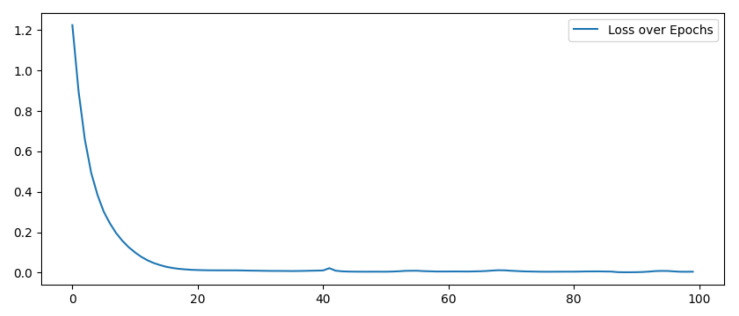
Losses during training over Epochs.

First of all, we trained the model to determine the best hyperparameters of the algorithm: the batch size has been set to 256 and epochs to 20, after the evaluation of the pattern of losses over the epochs (Figure 4).

**Figure 5:**
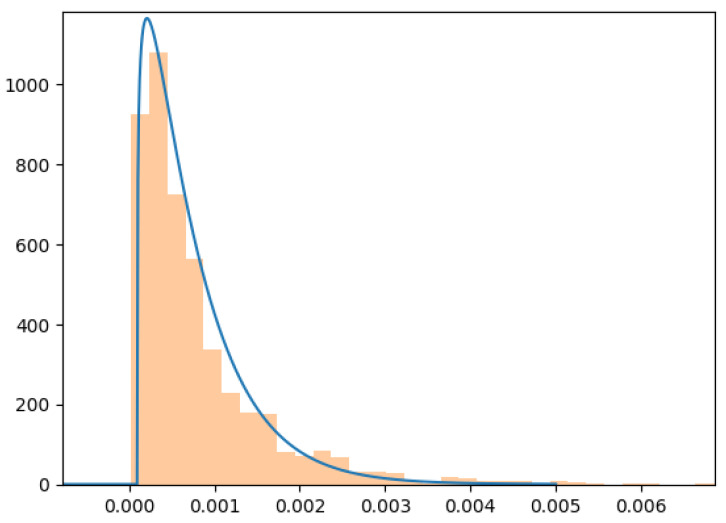
Distribution of reconstruction error over the training dataset.

Then we evaluated the reconstruction error over the training dataset. Results are shown in Figure 5. The error distribution can be approximated by a Gamma distribution depicted through a blue line. Consequently we chose the value of *E*, which allows maintaining the False Positive rate over the training dataset under $10^{6}$, by using the approximated gamma distribution. Since we chose to develop an anomaly detection algorithm, we have to set a threshold “a priori”, before evaluating the effective False Positive rate over the test dataset.

We tested a series of anomalies categorized in the following subsections.The same tests have been done by also using a traditional anomaly detection algorithm: One Class Support Vector Machine (OCSVM). The algorithm, implemented with the SciKit-Learn Library, has been set with a Polynomial Kernel. OCSVM has several applications, especially in the field of fault detection [29] while other Anomaly Detection algorithms such as Isolation Forest or Local Outlier Factory are more frequently used in other fields, such as Network Intrusion Detection Systems [30]. A comparison between the performance achieved with the autoecoder and the OCSVM are reported step by step for all anomaly conditions.

### 6.1. Violating Safe Operating Conditions

We tested the capability of the proposed solution to detect unsafe operating conditions, such as unsafe injected power (active or reactive), state of charge, currents, and voltages. In practice we wanted to check the ability of the algorithm to notice conditions in which the system is theoretically able to operate, but it is not supposed to do. For example, the power inverter may be sized to a 20% more or the nominal power, so that operating in this condition would not trigger any electrical protection, but it represents a possible risk.

The attack is conducted as follows:

1. Time interval 0–33.3 [s]: normal operation;
2. Time interval 33.3–76 [s]: the system injects 20% more active power with respect to the values seen in the training dataset;
3. Time interval 76–135 [s]: active power gradually increases by using steps of 10%;
4. Time interval after 135 [s]: the attack stops and the system returns to the initial condition.

**Figure 6:**
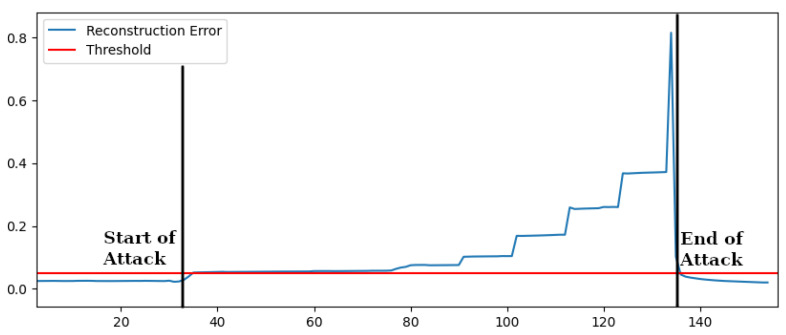
Reconstruction Error—Unsafe Power Injection.

Figure 6 shows the consequent reconstruction error over time in case of Active Power values over the ones seen in the training dataset. A variation of 20% with respect to the normal values seen in training is immediately detected. The reconstruction error is over the threshold. Additional variations make the case even more evident.

We can also notice in Figure 6 that if the attack ceases, the reconstruction error rapidly falls again under the threshold. Similar results have been obtained by testing data with similar variations concerning the range of normal functioning for reactive power, voltages, frequencies and state of charge. The results are summarized in Table 2 where the comparison with OCSVM is also reported. Concerning the violation of safe operating conditions OCSVM was also able to detect all the anomalies, showing performances comparable to the autoencoder-based anomaly detection.

### 6.2. Unusual Behavior

We tested the capability of the proposed solution to detect operating conditions in which each measure is within the limits seen in the training dataset but the behavior is unusual (i.e. not already seen). The attack is conducted as follows:

1. Time interval 0–31.25 [s]: normal operation;
2. Time interval 31.25–74 [s]: the injected reactive power falls to 0 while emitting active power (during training reactive power is positive and related to the injected active power);
3. Time interval after 74 [s]: the injected reactive power becomes negative.

An unusual behavior may be the consequence of a bad command sent to the power converter by exploiting vulnerabilities of the communication protocols.

**Figure 7:**
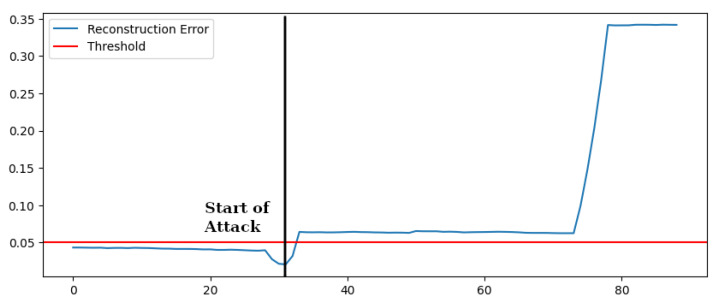
Reconstruction Error—Unusual Behavior.

In Figure 7 we show the reconstruction error, and it is clear how the attack is immediately detected by the autoencoder-based anomaly detection: as soon as the attack is started, the reconstruction error is above the threshold. After 74 [s], the impact of the attack is even more evident. The detection of negative reactive power injection is easy, since during the training the same value remains positive, but we included the analysis since such an attack might cause severe consequences on the grid. More interesting is the ability of the autoencoder-based algorithm to learn more complex correlations between measures and habits, differently from OCSVM.

Similar results have been obtained by testing the algorithm on different initial working conditions by varying the initial emitted power in all the usual range. Table 3 summarizes the result, also reporting the performance of the OCSVM scheme, which, in this case, does not satisfy because this type of attack is not detected.

### 6.3. Partial Attack

**Figure 8:**
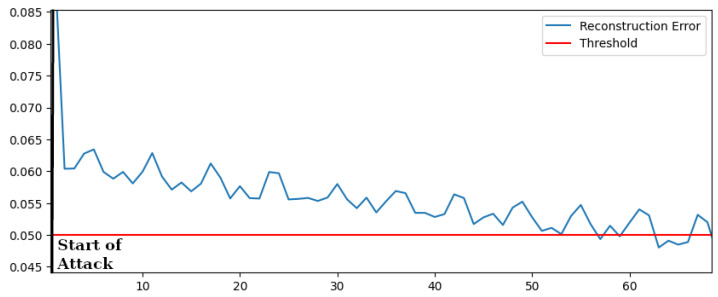
Reconstruction Error—Oscillating Power.

Commonly used protocols in the field of DERs, such as SV and GOOSE, periodically send the setpoint of power. If the attacker sends a fake command over the control network but he cannot intercept the right values, this may cause an oscillation of the injected power. We tested the capability of the algorithm to reveal such types of occurrences. The reconstruction error is shown in Figure 8 during an attack, started at time 0, which periodically sends a variation of 10% of active power.

It can be noticed that, even if the algorithm performs a static analysis of data, it is able to recognize the oscillation of the power thanks to the analysis of related physical parameters.

Similar results have been obtained for oscillation of reactive power. Results are summarized in Table 4), which also contains the performance of an OCSVM-based scheme, which again, fails to detect the attack.

### 6.4. Bad Data Injection

**Figure 9:**
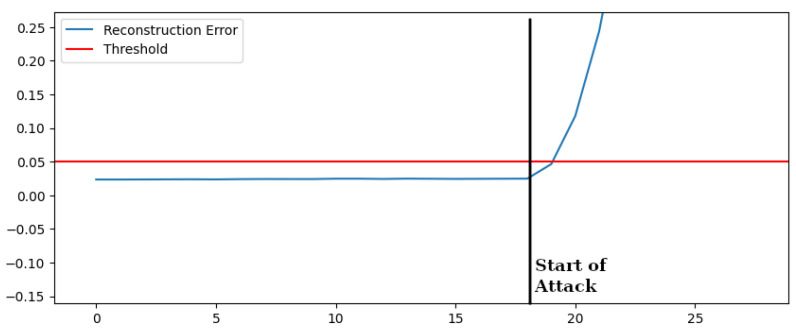
Reconstruction Error—Bad Data Injection Voltage.

We make the hypothesis of performing a Bad Data Injection attack by modifying a small subset of measures (even one) in order to create a state vector that represents an unfeasible working condition. In Figure 9 we show the reconstruction error of the test dataset containing a bad data injection over the voltages of the three phases. The attack is carried on by modifying the value of the voltage of a small percentage every second, as follows:

1. Time interval 0–18 [s]: normal behavior;
2. Time interval after 18 [s]: the attack starts. The value changes of 1% with respect to the current value of each second.

The autoencoder-based algorithm rapidly detects the attack. Since the algorithm performs a static analysis of data, the reconstruction error increases over time, independently of the bad data injection changing rate. The same results have been obtained for Bad Data Injection over Powers, frequencies, and other parameters as reported in Table 5 where also the performance of OCSVM is shown. OCSVM, in this case, was able to detect some attacks but was unable to detect attacks in which the variations of the measures are not significant in absolute terms. In the case of bad data injection of voltages, as shown in Figure 9, OCSVM detected the attack later than the proposed solution.

## 7. Discussion

The autoencoder-based proposed solution can detect a wide set of anomalies and also limits false positives. Two main advantages can be identified if compared with the OCSVM solution already at the state of the art:

- The autoencoder-based algorithm performs a static analysis of data. This result reflects positively in the capability to identify attacks even when a small variation over time is measured, differently from the OCSVM-based solution;
- The autoencoder-based algorithm detects some types of anomalies better. The One Class Support Vector Machine-based solution performed worse due to the reduced capability to learn correlation between measures.

The capability to detect Bad Data Injection attacks even if conducted by a slow modification of the considered measures is a very interesting feature of the proposed algorithm. It has been shown that an attacker can exploit the configuration of a power system and launch such attacks to successfully introduce arbitrary errors into certain state variables while bypassing existing techniques for bad measurement detection [31]. A static analysis of data is immune to such threat by design.

Nevertheless, the proposed solutions shows some limitations. For example, a particularly dangerous attack could be a bad data injection over the State of Charge of the battery, resulting in bad decisions taken from the higher-level controller. The present version of the autoencoder-based algorithm is still unable to learn a highly precise estimation of the state of charge and is inefficient for the detection of sophisticated attacks over this measure. More complex neural network architectures should be investigated to address this issue. Recurrent Neural Networks have been used in the literature to obtain a precise estimation of the State of Charge of BESS, outperforming traditional methods [32]. This observation suggests that some form of regression model can be used to build an anomaly detection algorithm also tackling bad data injection over the State of Charge.

## 8. Conclusions

This paper analyzed a typical architecture of a storage system within a microgrid, discussing possible vulnerabilities and evaluating risk scenarios, and proposed an anomaly detection algorithm based on a neural network architecture called autoencoder. The proposed solution was able to detect a series of attacks in a simulated environment, outperforming a traditional One Class Support Vector Machine-based anomaly detection algorithm, used as the comparison. The proposed algorithm can be applied both to SCADA systems, like in a microgrid, and to Cloud Applications, which can monitor a large number of geographically dislocated generators by substituting the supervision of a human operator. The obtained results are really promising, but future development should include the evaluation of more complex neural network architectures with the aim to include the detection of more sophisticated attacks. 

## Figures and Tables

**Table 1:** List and description of the features.

Feature	Symbol	Description
$X_{1}$	SOC	battery state of charge estimated by the BMS
$X_{2}$	$V_{cells}$	voltage measured at the terminals
$X_{3}$	$I_{cells}$	current emitted by cells array
$X_{4}$	$V_{d}c$	average voltage in the DC link
$X_{5}$	$V_{a}$	voltage of phase a (AC side)
$X_{6}$	$V_{b}$	voltage of phase b (AC side)
$X_{7}$	$V_{c}$	voltage of phase c (AC side)
$X_{8}$	$I_{a}$	current of phase a
$X_{9}$	$I_{b}$	current of phase b
$X_{10}$	$I_{c}$	current of phase c
$X_{11}$	$f_{a}$	frequency of phase a
$X_{12}$	$f_{b}$	frequency of phase b
$X_{13}$	$f_{c}$	frequency of phase c
$X_{14}$	$THD_{a}$	total harmonic distortion of voltage on phase a
$X_{15}$	$THD_{b}$	total harmonic distortion of voltage on phase b
$X_{16}$	$THD_{c}$	total harmonic distortion of voltage on phase c
$X_{17}$	Qset	last reactive power setpoint sent by the SCADA controller
$X_{18}$	Pset	last active power setpoint sent by the SCADA controller
$X_{19}$	*Q*	reactive power emitted by the inverter
$X_{20}$	*P*	active power emitted by the inverter

**Table 2:** Tests—Violating Safe Operating Conditions.

Attack	Description	Autoencoder	OCSVM
Bad Set Point Active Power	±20% of normal values, ramp	yes	yes
Bad Set Point Reactive Power	±20% of normal values, ramp	yes	yes
Bad Set Point Voltage	±5% of normal values, ramp	yes	yes
SOC over normal limits	±20% of normal values	yes	yes

**Table 3:** Tests—Partial Attack.

Attack	Description	Autoencoder	OCSVM
Variation of Ratio between Active and Reactive Power			
All Range of Active Power	±30% of normal values	yes	no

**Table 4:** Tests—Partial Attack.

Attack	Description	Autoencoder	OCSVM
Active Power Oscillation	±10% of normal values	yes	no
Reactive Power Oscillation	±10% of normal values	yes	no

**Table 5:** Tests—Violating Safe Operating Conditions.

Attack	Description	Autoencoder	OCSVM
Voltage, 1 phase	±1% variation per second	yes, after 1 s	yes, after 5 s
Voltage, 3 phase	±1% variation per second	yes, after 1 s	yes, after 5 s
Frequency, 1 phase	±1% variation per second	yes, after 1 s	yes, after 5 s
Frequency, 3 phase	±1% variation per second	yes, after 1 s	yes, after 5 s
SOC	±20%, step	yes	no

## Data Availability

Not applicable.

## Abbreviations

The following abbreviations are used in this manuscript:

DER	Distributed Energy Resources
RES	Renewable Energy Sources
BESS	Battery Electrical Storage System
BMS	Battery Management System
PCS	Process Control System
HMI	Human Machine Interface
EMS	Energy Management System
SOC	State Of Charge
AFE	Active From End
ML	Machine Learning
NN	Neural Network
SCADA	Supervisory Control and Data Acquisition
DCS	Distributed Control System
MIT	Man In the Middle
